# A Systematic Review of the Sustainable Campus Concept

**DOI:** 10.3390/bs12050130

**Published:** 2022-04-29

**Authors:** Agus Sugiarto, Cheng-Wen Lee, Andrian Dolfriandra Huruta

**Affiliations:** 1 Department of Management, Faculty of Economics and Business, Satya Wacana Christian University, 52-60 Diponegoro Rd, Salatiga City 50711, Indonesia; 2 Department of International Business, College of Business, Chung Yuan Christian University, 200 Zhong Bei Rd, Taoyuan City 32023, Taiwan; chengwen@cycu.edu.tw; 3 Ph.D. Program in Business, College of Business, Chung Yuan Christian University, 200 Zhong Bei Rd, Taoyuan City 32023, Taiwan; g10804610@cycu.edu.tw

**Keywords:** sustainability, Sustainable Campus, green behavior, higher education

## Abstract

Higher education institutions’ contributions to environmental conservation are manifested in their commitments to develop Sustainable Campuses. Numerous studies have investigated higher education institutions’ efforts to create Sustainable Campuses. Many studies on Sustainable Campuses have been completed partially. The analysis is carried out on the basis of practices at various campuses around the world. However, a thorough analysis of Sustainable Campuses has so far not been carried out. This is evidenced by the lack of publications on journal database portals related to Sustainable Campuses which are carried out in a systematic literature review. To address this gap, this study provides a systematic and comprehensive review of the literature on Sustainable Campuses. The purpose of this article is to identify various dimensions of implementing Sustainable Campuses from various countries. We use the qualitative systematic review method with the meta-aggregation approach in this study. The results of this study indicate that Sustainable Campus development activities are classified into three aspects such as behavioral, learning and educational tools, and physical facilities. Further, each dimension has several strategies and programs and actions performed by global higher education institutions. The results are also expected to be a motivator and reference for campuses to contribute to environmental conservation through Sustainable Campus programs. The various dimensions of a Sustainable Campus that are mapped out in this research can be used as a reference for realizing a Sustainable Campus for every university campus in various countries.

## 1. Introduction

The global commitment to environmental preservation increases due to the current global warming phenomenon. Global warming and climate change are not illusions but real environmental problems to be resolved immediately. Various anomalous natural phenomena such as an uncertain climate, lengthy and extreme heat, high rain intensity, storms, and tornados are arguably related to global warming. Several studies demonstrate increased CO_2_ greenhouse gas levels in the atmosphere due to increased human activities on earth, including household activities (institutions/offices/hospitals/schools/campuses) and industrial and transportation activities.

Environmental problems and their mitigation efforts are increasingly complex, including various broad aspects, while human understanding about environmental issues remains far from perfect. Environmental problems commonly require synergies from all public elements, including *civitas academica*. It takes strong commitments from all parties to reduce the environmental degradation rate. Currently, these commitments are reflected in various actions and programs in several life dimensions. Business organizations implement their environmentally friendly commitments in various programs, including green businesses [1,2], sustainable offices [3], green production [4,5,6], green marketing [7,8], green human resource management [9,10], green supply chain management [11,12], and other concepts related to environmental friendliness. Environmentally friendly concepts are developed as business organizations’ commitments to preserve the natural environment. Meanwhile, educational organizations develop green campuses, sustainable education, sustainable schools, and Sustainable Campuses as environmentally friendly concepts [13,14].

A Sustainable Campus is defined as an environmentally oriented campus that integrates environmental science into its policies, management, and scholarly activities [15]. A Sustainable Campus also represents the implementation and integration of environmental science into all managerial aspects and the best practices of sustainable development [16]. Many universities worldwide have shown their commitments to implement the Sustainable Campus concept. The Sustainable Campus concept needs to be implemented because various studies show that the stakeholders of universities that implement the Sustainable Campus are significantly more satisfied and have better perceived life quality than those from non-implementing universities [17]. Besides, Sustainable Campus implementation also helps energy conservation and efficiency [18,19].

Several global universities have also implemented the Sustainable Campus. For example, the University of Southern Santa Catarina has developed global partnerships to encourage scientific production and sustainable practices to be an example of green campuses in southern America [20]. According to the STARS (Sustainability Tracking Assessment and Rating System), Stanford, one of the best greenhouses, has implemented Sustainable Campuses in three steps. First, it introduces academicians to the detailed needs of supplies, energy, water, land, waste, management, food, life, buildings, and campus development transportation. Second, it uses STARS to make comprehensive and sustainable evaluations of Stanford. Lastly, it discusses the development of the relationship between Stanford and its local communities [21]. Besides, a study in Malaysian universities finds that Malaysian higher institution educations currently implement green practices in their campuses to support sustainability [22]. Further, Jordan University of Science and Technology also initiates efforts to change its campus into a green, energy-saving, and low-carbon campus by following an action-oriented strategy [23].

The University of Indonesia initiates the greenhouse rating program labeled as UI Sustainable Metric World University Rankings to map global universities’ performance in environmental friendliness. This program aims to conduct online surveys of worldwide campuses to investigate their sustainable programs and policies by requiring participants to participate in the following years [24,25,26]. Besides the UI Sustainable Metric World University Rankings, other green rating programs include the DEA-Greenmetric [27], STAR [21], Environmental Management System (EMS) ISO 14001, and United Nations Environment Program (UNEP) [28,29].

The presence of several rating agencies in universities’ environmental friendliness arguably benefits universities’ sustainability. Atici et al. proposed higher education institutions’ environmentally friendly commitments and practices reflect their rankings and reputations, and environmental protection can be their competitive advantage [30]. A thorough search of many studies on Sustainable Campuses indicates several strategic aspects implemented by campuses to achieve Sustainable Campuses, namely behavioral aspects [31,32,33,34,35,36,37,38,39], learning instrument aspects [40,41,42,43,44], and campus physical facilities [23,45,46,47,48,49,50,51,52].

The contribution of the world community in realizing the Sustainable Development Goals through commitments to environmental conservation in recent years has attracted a lot of attention from academics and practitioners around the world, likewise for the higher education community. Quite a number of studies on the application of the concept of a Sustainable Campus have been carried out in various countries. Many studies on Sustainable Campuses have been completed partially. The analysis is carried out on the basis of practices at various campuses around the world. However, a thorough analysis of Sustainable Campuses has so far not been carried out. This is evidenced by the unavailability of publications on journal database portals related to Sustainable Campuses which are carried out in a systematic literature review. To address this gap, this study provides a systematic and comprehensive review of the literature on Sustainable Campuses.

Global universities have implemented the environmentally friendly concept with varying success levels, standards, and implementation strategies. Thus, this article aims to analyze various strategies used to implement the Sustainable Campus concept. In particular, our research problems are: (1) What strategies in the behavioral dimension are implemented by higher education institutions to achieve a Sustainable Campus? (2) What strategies in the learning instrument dimension are implemented by higher education institutions to achieve a Sustainable Campus? and (3) What strategies in the campus physical facilities dimension are implemented by higher education institutions to achieve a Sustainable Campus?

This study seeks to identify and understand strategies in the behavioral, learning tool, and physical infrastructure dimensions of higher education institutions in implementing and achieving Sustainable Campuses through a literature review. It is expected that this study illustrates and informs comprehensively on current strategies to achieve Sustainable Campuses.

## 2. Research Methods

We use the qualitative systematic review method with the meta-aggregation approach in this study. Systematic review analyzes all studies relevant to certain research questions, topics, or phenomena of interest [53]. The meta-aggregation approach of the research topics is then elaborated further into certain themes to produce an analytical framework. Next, for each theme, the study searches for relevant articles and compares and summarizes these articles. In the meta-aggregation approach, the synthesis results are the “aggregate” of various studies on relevant themes. Further, synthesis aims to answer research questions by summarizing various studies [54].

**Figure 1:**
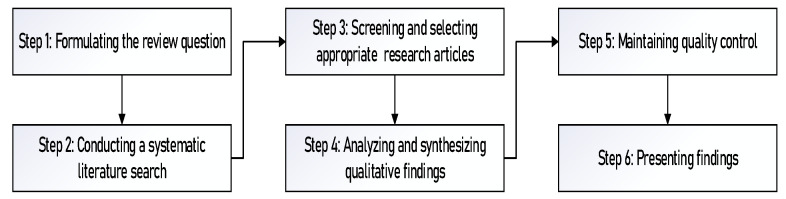
Phases of qualitative systematic review.

Following Francis-Baldesari, we organize our research into the following six phases [55]. The phases related to qualitative systematic review is presented in Figure 1.

The number of articles used as the basis for a systematic review is 100 article titles. This number was filtered from the portal database with the keyword “sustainable campus” title, which found 9695 titles. The number of titles was obtained from Portal: EBSCOhost Research Databases; tracked using Keywords: Sustainable campus; in Source Types: Academic Journals; Data base: Academic Search Complete; Journal category: peer reviewed; and published in the range of 2011 to 2021.

**Figure 2:**
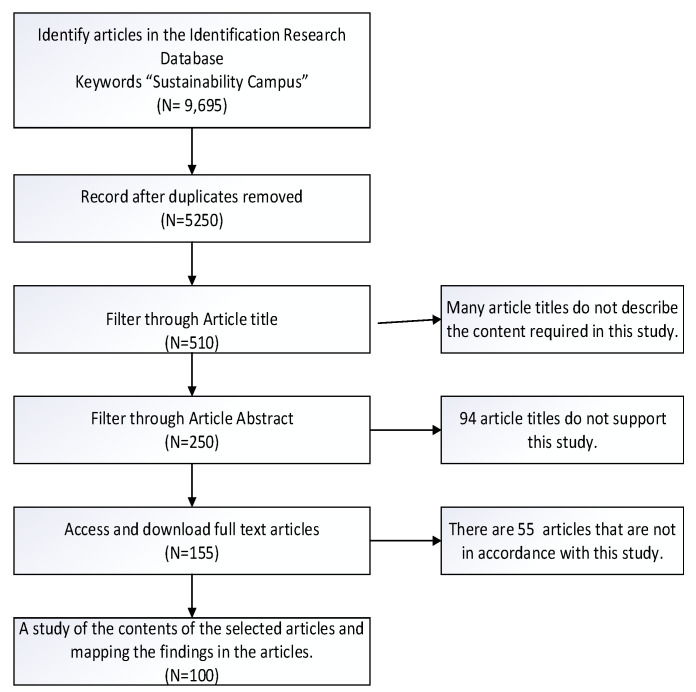
Searching strategy and study selection process.

While the process of identifying and searching for article selection participants is carried out through the following process in Figure 2.

## 3. Results and Discussion

The systematic review of current articles on Sustainable Campuses is based on three research problems: (1) strategies in the behavioral dimension implemented by higher education institutions to achieve Sustainable Campuses, (2) strategies in the learning instrument dimension implemented by higher education institutions to achieve Sustainable Campuses, and (3) strategies in the campus physical facilities dimension implemented by higher education institutions to achieve Sustainable Campuses.

### 3.1. Behavioral Dimension

#### 3.1.1. Strategy 1: Strengthening Leaders’ Commitments

Leaders’ roles are crucial in building sustainable organizations through the implementation of the environmentally friendly concept. Singh et al. argue that leadership plays an important role in affecting human resource management and eventually predicts organizational green innovations [37]. Other researchers also analyze the role of leaders in building the environmental friendliness concept in organizations [31,56]. They found similar results, that leaders’ descriptive environmental norms and their pro-environmental leadership and behavior play a vital role in organizational greening.

The roles of universities are also vital in achieving a Sustainable Campus. Ribeiro et al. proposed that the leadership factor plays a significant role in realizing a Sustainable Campus [20]. Similarly, Fissi et al. also showed universities have implemented clear strategies and well-structured initiatives to implement sustainable practices [39]. Further, rectors fully support the institutions to become greener. Other studies also analyze the roles of leaders in building sustainable and green campuses [16,57].

The above arguments indicate that university leaders play crucial roles in building Sustainable Campuses. Their roles can be realized in their commitments to create an environmentally friendly atmosphere, initiate and motivate the implementation of the Sustainable Campus program, and provide policy instruments oriented towards Sustainable Campuses. Besides, they need to become good commanders in the implementation process of Sustainable Campus programs. *Civitas academica* also need to exhibit exemplary environmentally friendly behaviors.

#### 3.1.2. Strategy 2: Building Campus Communities’ Involvement in Environmentally Friendly Activities

In general, environmentally friendly activities act as an employee participation framework to strengthen organizations’ environmentally friendly policies. Pardal et al. argued higher education institutions play a crucial role in encouraging sustainability. Sustainability has been considered as a teaching component, research, innovation, and a social learning process within or outside academic activities [58]. Martinez-Buján et al. presented the importance of universities’ sustainable social dimension [36]. Choi et al. demonstrated that students who have taken sustainability-related courses or participated in sustainability activities have more knowledge on green campus strategies than those who do not [34]. Meanwhile, Azar and Al Ansari revealed several factors, including respondents’ demographic characteristics, control over building systems, and motivational boosters’ (e.g., financial, social, and environmental) heavily energy-saving needs and actions through effective human-centered energy conservation strategies [59]. The factors include psychological needs, physical facilities, personal motivation, public perception, and policies [32]. In this respect, Fachrudin and Fachrudin documented that awareness, attitude, subjective norms, behavioral control, and intention are the main indicators of green behavior [38].

To involve campus stakeholders more, Wimala recommend that campus management enhance socialization and educational programs to their staff and students, increase institutional commitments, and increase research and collaboration on environment-related issues [33]. The UI GreenMetric assessed universities based on their commitments and actions on greening activities and environmental sustainability [35]. Besides the UI GreenMetric, the UNEP (United Nations Environment Programme) ranking program is also worth considering [28].

The following are several activities that are needed to implement this strategy: (1) internalizing environmentally friendly concepts on all *civitas academica*, (2) organizing environmentally friendly extracurricular programs, (3) developing an environmentally friendly culture in daily activities, (4) developing environmentally friendly behavioral control systems, and (5) participating in Sustainable Campus ranking programs, including the UI GreenMetric, DEA-Greenmetric, and other ranking programs. Table 1 presents a summary of several strategies and programs in the behavioral dimension to realize a Sustainable Campus.

### 3.2. Learning Tool Dimension

#### 3.2.1. Strategy 3: Creating and Implementing Sustainable Curriculums

Creating environmentally friendly curriculums is a strategy to develop Sustainable Campuses. Menon and Suresh recommend integrating sustainability into teaching and learning and other educational aspects [43]. Revelli established that Sustainable Campus implementation requires environmentally friendly curriculums [40]. Students’ learning experience is also crucial in implementing environmentally friendly concepts in the learning process and curriculums [42]. Similarly, Hays and Reinders also emphasized the importance of ecological thoughts and systems and self-sufficiency as the tools and objectives of sustainable education and learning [44]. Successful integration of sustainable principles and methods into technical curriculums requires systemic changes in current educational approaches. Students need to be equipped with cognitive skills and high-level critical thinking to facilitate transitions toward a low-carbon economy instead of theoretical knowledge of sustainable development. Gress and Shin have investigated the implementation of green curriculums in Korea [41].

Thus, it can be concluded that creating and implementing curriculums is one of the implementation strategies of Sustainable Campuses. Several activities to support this strategy include developing environmentally friendly curriculums and incorporating environmentally friendly and sustainability values into course contents.

#### 3.2.2. Strategy 4: Adopting Environmentally Friendly Technology in Learning Processes

Universities become sustainable by considering the use of advanced technology and students’ preparedness. Technology adoption enhances universities’ opportunities to go sustainable [60]. Yolcu and Han also analyzed the use of technology in the learning process and found that students use the internet for their learning objectives. Students from three universities exhibited similar levels of technology use in their learning processes [61]. A similar idea suggests developing sustainable e-learning frameworks to offer sustainable learning quality through the technological, application, sustainable development, and learning principle perspectives [62]. Similarly, Naveh and Shelef found that students extensively use various technologies to learn [63].

Sustainable e-learning helps the higher education sector increase the supply of innovative and creative graduates while reducing costs by using resources more efficiently. A very promising way to offer innovative learning environments is through e-learning. This argument leads to how to sustain economic development and education and how e-learning plays a role in achieving and preserving sustainability [64]. The use of technology in the smart classroom system based on live webcasts is facilitated through several stages, namely: (1) system design, (2) system creation, and (3) system testing. Developed systems enable learning interactions in physical and virtual classes in different locations [65]. Besides, Khlaisang and Songkram proposed that digital media enable learning processes to be held anytime and anywhere. The use of cellular equipment and 3-D virtual classrooms offer integrated environments for effective learning [66].

#### 3.2.3. Strategy 5: Developing Paperless Offices

Academic administrative offices are one of the supporting functions in campus activities. This unit offers academic administrative services for students and lecturers. One of the office units’ supports in building Sustainable Campuses is developing paperless offices. Paper is every office’s main medium of supplies. Reduced paper use is an environmentally friendly behavior. The environmentally friendly office concept is also commonly known as the green office or eco-office concept [67,68]. Sboui et al. investigated the implementation of paperless offices [69].

Universities can implement their initiatives to achieve Sustainable Campuses through paperless offices by using IT. They can make letters and manage their files digitally or electronically. Some other researchers have examined the implementation of the eco-office concept in universities through paperless offices [67,68,69,70,71,72,73,74].

Ugale et al. illustrated how universities realize effective document processing by scanning, marking, and indexing for effective data gathering with Optical Character Recognition (OCR) and indexing [71]. Next, Indrajit et al. described the development of the paperless office concept as a mediator between traditional and digital learning processes [72]. Meanwhile, Genesis and Oluwole explained the paperless process in inputting the senate process, staff assessment, student registration, examinations, and bulletin production through full automatization. Universities can achieve paperless school systems faster by greater support from management and decision makers. Consequently, information can be accessed efficiently (speed), effectively (better information source), and in an environmentally friendly way [73]. Similarly, Onwubere underlined the importance of ICT implementation in universities’ administration [74].

The above arguments conclude that adopting environmentally friendly learning technology in the learning process is important in achieving Sustainable Campuses through IT implementation and e-learning applications that enable virtual learning. Further, electronic books (e-books) are another environmentally friendly learning medium because they do not need paper as with conventional books. Other learning technologies are audio and video media. Table 2 provides a summary of several strategies and programs in the learning tool dimension for achieving a Sustainable Campus:

### 3.3. Physical Facility Dimension

#### 3.3.1. Strategy 6: Evaluating and Revitalizing Environment-Based Campus Masterplans

Physical planning offers opportunities to integrate ecological priorities into universities’ missions. Hence, it is recommended that universities preserve their ecology through spatial and strategic planning [75]. Batalla and Sánchez suggested that universities need to have campus planning through commitments commonly agreed to by stakeholders operationalized into several concrete phases into master plans [76]. Several studies emphasize the importance of strategic planning in achieving Sustainable Campuses [51,52,77,78,79].

Sustainable planning should be made based on campuses’ green building designs; how much a Sustainable Campus building will cost; how sustainable planning affects energy use during an academic year; and the direct benefits of the campus’s sustainable design and planning for faculties, students, staff, administrators, the environment, and the public [80]. Further, the campus’s environmental masterplans need to consider smoke-free zones. Leal Filho et al. mentioned the formal sustainable development policies, as indicated by various environmental protection policies or procedures [81].

Physical planning provides opportunities to integrate ecological priorities into universities’ missions [75]. In this regard, the Sustainable Building Council Indonesia (GBCI) set the sustainable building criteria with one evaluation aspect for Energy Efficiency and Conservation (EEC) that is closely related to efficient energy consumption [82]. In this dimension, the evaluation of the Sustainable Campus’s development is a crucial tool to evaluate higher education institutions’ responses to national and environmental policies [83].

In this strategy, universities can make several activities or programs in formulating environmentally oriented visions and missions, developing strategic environmentally friendly campus development plants, making environmentally friendly building-standard designs, designing sustainable infrastructure, designing smoke-free zones (including producing, selling, advertising, and promoting tobacco products), designing open green space for parks and campus reforestation efforts, and evaluating Sustainable Campus programs.

#### 3.3.2. Strategy 7: Improving Water Quality and Usage Efficiency

The green campus is a concept applied by campuses with ecologically oriented policies [84]. The program’s water reuse and waste management also need to be developed to improve universities’ water quality. A study in the Jordan University of Science and Technology (JUST) revealed that daily per capita water consumption is about 56 L, about a third of the water consumption of a student in a U.S. institution [23]. Meanwhile, Fahrianto et al. revealed that a drinking-water provision is crucial to support the green campus program [85].

Some studies illustrated several water resource conservation practices. For example, Peng et al. analyzed how the University of Tianjin develops a green campus using rain water and a sustainable water circulation system. In its campus planning, the University of Tianjin develops a multilevel rainwater collection, use, and disposal regime with flood discharge and rainwater resource use as its main priorities [49]. Some other researchers also examine campuses’ water resource management and conservation practices [86,87,88].

Hence, improving clean water usage efficiency and drinking water quality is a strategy to implement Sustainable Campuses. This strategy can be achieved by revitalizing clean water networks, metering the water supply in each building, reusing wastewater, improving the surface water’s quality, and collecting, managing, and conserving rainwater.

#### 3.3.3. Strategy 8: Improving the Energy-Use Efficiency of Electricity

The development and promotion of the sustainable green concept are significant steps to change academic campuses into energy-efficient and environmentally sustainable communities [19,59]. Faghihi et al. highlighted that reducing energy use is crucial to improve campus sustainability through increased infrastructure efficiency and conservation [89].

In this regard, Tan et al. showed the rapid and large-scale development of energy and resource-efficient campuses in China, especially through the implementation of energy-saving technologies and campus energy management [90]. Revelli illustrated that the Lake Park High School District 108 in Roselle, Illinois, is expected to provide clean and renewable energy to save energy use by 5.1 million dollars [40]. Further, Suwartha et al. exposed that the initial phase of renewable energy development still faces challenges [91]. Campuses can improve their energy efficiency practices by using solar light as an energy source in their environments [47,48,92,93].

Higher education institutions implement their electricity energy-saving strategy by reconfiguring networks, metering each faculty or work unit, automating electricity energy use in classes, and regulating public lights. Universities’ internal policies have stipulated the regulation of air-condition temperatures and the replacement of old electrical equipment with energy-saving ones. Several interdisciplinary studies on renewable energy (including solar lights as an energy source on campuses) have also been conducted as one of the leading research topics in many universities.

#### 3.3.4. Strategy 9: Integrated Trash Management

Waste management is a dimension to achieve Sustainable Campuses. A study documents that littering and open-trash-disposal behaviors are still common. Besides, open trash burning is often considered a common way to manage campuses’ large-scale waste. The research also shows that only 49.5% of students express serious concerns about trash management practices [45]. Next, Ifegbesan confirmed that although students are directed positively toward innovative ways to overcome universities’ trash management, they show significant differences in awareness and disposition based on sex, age, academic level, and faculty status [45].

Waste management initiatives positively affect public attitudes towards resources, waste management, and awareness of reducing waste. However, pilot projects increase campuses’ overall recycling rate from 10 to 12% [94]. Smyth et al. showed that the Prince George campus produces between 1.2 and 2.2 metric tons of waste per week, more than 70% of which are diverted through waste reduction, recycling, and composting [95]. Further, Abu Qdais et al. pointed out that the average increase in trash generation at the Jordan University of Science and Technology (JUST) is lower and better than related data at other universities in both developing and developed countries [23].

Khandelwal also demonstrated trash management practices by illustrating their potential benefits by producing biogas from organic waste and optimizing resources through the 3R concept (reduce, reuse, and recycle) for glass, metal, and others. Campuses’ trash management is also related to existing rules. Higher education institutions’ trash management models are often subject to existing bureaucratic controls and regulations [46]. Tiew et al. concluded that their university’s good trash management system is a good example for other universities because it positively affects trash recycling management in campus environments [50].

Universities can implement their trash management strategy through the composting center program to manage their trash. Integrated trash management and implementing policies related to independent trash management within the campus environment are also parts of campus trash management. Universities can organize public service activities for these programs in cooperation with surrounding communities to use recycled materials.

#### 3.3.5. Strategy 10: Developing Environmentally Friendly Internal Campus Transportation

Universities need to make integrated managerial actions in greening their transportation and commuter parking in their campuses, identifying and measuring opportunities to make transitions to a more sustainable future, and orienting themselves to improve public welfare and reduce environmental impacts [96]. Kaplan showed the low levels of students’ sustainable transports around campuses as indicated by very low cycling activities [97]. Abu Qdais et al. found that the per capita carbon emission of the JUST campus is 1.33 tons of CO_2_ equivalent, smaller than other universities worldwide [23].

Bond and Steiner stressed the University of Florida has established sustainable, long-term cooperation with the local transit systems in Gainesville, Florida, to develop a Sustainable Campus [98]. It takes supporting infrastructure to develop environmentally friendly transportation on campuses [99]. Besides, managing campus transportation can be a model for the public in general if the general transportation systems in campuses are developed efficiently [100,101].

A strategy to achieve Sustainable Campuses is developing environmentally friendly and mass transportation infrastructure to serve routes to campuses nearby and other areas. Thus, it requires integrated research on designing environmentally friendly transportation devices, planning transportation management systems, building the supporting facilities of environmentally friendly mass campus transportation, cooperating with other related organizations and industries, planning transportation devices, and developing supporting infrastructure and facilities. Table 3 summarizes several strategies and programs for re-creating a Sustainable Campus in the physical facility dimension.

## 4. Conclusions

Public awareness of environmental protection is increasing in line with shared commitments through the Sustainable Development Goals (SGDs). One of the important dimensions in the goal of sustainable development is environmental sustainability. This is marked by increasing their contribution to environmental conservation efforts. Higher education, as a human-resource-development institution, has a contribution to environmental conservation efforts. Achieving a Sustainable Campus is a manifestation of the contribution of universities to environmental preservation. Efforts to realize a Sustainable Campus by universities in various countries have been portrayed through a number of publications of research results. Through research and publications on Sustainable Campuses, the variety of strategies and ways that campuses in various countries seek to create a Sustainable Campus can be identified.

Our systematic review of several articles in international journals concerning Sustainable Campuses indicates that Sustainable Campus development activities are classified into three aspects or dimensions: behavioral, learning and educational tools, and physical facilities. Each dimension contains several strategies that are used by various higher education institutions to create a Sustainable Campus. Furthermore, each strategy identified various programs and actions in realizing a Sustainable Campus.

In the behavioral dimension, there are two strategies used, namely strengthening leadership commitment, while the second strategy is building green engagement. In the dimension of the learning tool, three strategies were identified. The first strategy is developing and implementing a sustainable curriculum. The second strategy is adopting environmentally friendly technology in learning, and the third strategy is developing a paperless office. In the physical facility dimension, several strategies have been mapped out. The first strategy is evaluating and revitalizing the environment-based campus master plan, the second strategy is improving water quality and use efficiency, and the third strategy is improving electricity energy use. The next strategy is integrated trash management, and the last strategy is developing environmentally friendly campus transportation.

The results of this systematic literature review are expected to be a driving force and reference for higher education institutions to contribute to environmental conservation through efforts to create a Sustainable Campus. The three dimensions of a Sustainable Campus and the ten strategies mapped out in this research can be used as a reference for realizing a comprehensive Sustainable Campus for every university campus in various countries. Thus, the author hopes that many campuses will succeed in realizing Sustainable Campuses, so that they can contribute to achieving the Sustainable Development Goals (SDGs).

Our research was limited to reviews of academic articles obtained from online databases which contained the words “Sustainable Campus” in the search key titles. In addition, only papers from peer-reviewed journals were used. Thus, future empirical research could consider other related books regarding the topics to enrich the generalizability of the key findings. To measure actual Sustainable Campus’ sustainability, it might be important to explain the length of time to operate the Sustainable Campus initiatives. Hence, a longitudinal study is highly recommended to enhance research validity. Other software could be considered in future research to increase the statistical inference of review paper analyses, such as Comprehensive Meta-Analysis (CMA), Preferred Reporting Items for Systematic Reviews and Meta-Analysis (PRISMA), etc.

## Figures and Tables

**Table 1:** Strategy in the behavioral dimension.

Strategy	Program/Action
Strategy 1: Strengthening Leadership Commitment	Cultivating and developing the commitments of universities’ leaders to environmentally friendly behaviors [31,37,56,57].
	Motivating higher education institutions’ leaders to become initiators and motivators in Sustainable Campus programs [31,37,56,57].
	Providing a set of policies oriented towards Sustainable Campuses [16,20].
	Encouraging higher education institutions’ leaders to become good commanders in the implementation of Sustainable Campus programs [31,37,56,57].
	Motivating higher education institutions’ leaders to provide exemplary environmentally friendly behaviors for campus communities [31,37,56,57].
Strategy 2: Building Green Engagement	Internalizing the environmentally friendly concept in all *civitas academica* [32,33,36,58].
	Organizing environmentally friendly extracurricular programs [36,58].
	Developing an environmentally friendly culture in daily activities [33,58]. Developing environmentally friendly behavioral control systems [38,59].
	Participating in Sustainable Campus ranking programs [27,28,29,35].

**Table 2:** Strategies in the learning tool dimension.

Strategy	Programs/Actions
Strategy 3: Developing and Implementing Sustainable Curriculums	Developing and implementing environmentally friendly curriculums [40,41,42,43,44]. Incorporating environmentally friendly and sustainability values in course contents [40,41,42,43,44]. Developing environmentally friendly learning methods [40,41,42,43,44].
Strategy 4: Adopting Environmentally Friendly Technology in Learning	Using e-learning applications that enable virtual learning processes [61,62,63,64].
	Using audio and video learning technology [60,61,62,63,66].
	Using online classes to encourage collaboration and involvement to motivate students [65,66].
Strategy 5: Developing Paperless Offices	Creating and distributing mail digitally [67,68,69,74]. Scanning paper documents into digital forms [71,72]. Managing office files electronically [67,72,73,74]. Managing campus bulletins digitally [72,73]. Administering online student admissions [72,73]. Digital or online performance evaluation of lecturers and staff [73,74].

**Table 3:** Strategies in the physical facility dimension.

Strategy	Programs/Actions
Strategy 6: Evaluating and Revitalizing Environment-based Campus Masterplans	Formulating visions for environmentally oriented missions [51,75].
	Making strategic plans for environmentally friendly campus development [51,52,75,76,77,78,79].
	Designing environmentally friendly building standards [80,82].
	Planning sustainable infrastructure designs [80,82].
	Developing smoke-free zones, planning open green zones for parks and campus reforestation efforts [80,81].
Strategy 7: Improving Water Quality and Use Efficiency	Improving the use efficiency of clean water [23,85,102]. Revitalizing clean water networks and metering water supplies in each building [23,49,85]. Initiating water reuse programs [23,49,75]. Improving surface water quality [85,86,87]. Managing and conserving rainwater through infiltration wells [49,86,87,88].
Strategy 8: Improving Electricity Energy Use	Energy efficiency by: reconfiguring the electricity network, metering electricity, automating and regulating energy, internal policies that regulate air condition temperature, replacement of old electrical equipment with energy-saving new ones [59,89,90]. Using solar light as an energy source in campus environments [19,40,47,92,93].
Strategy 9: Integrated Trash Management	Developing composting center programs to manage trash from campus activities, by reducing, reusing, and recycling campus waste [46,50,94,95,103].
	Preparing integrated trash management and making policies related to independent trash management [23,45,46,94,103].
Strategy 10: Developing Environmentally Friendly Campus Transportation	Developing environmentally friendly mass transportation [23,96,97,100,101].
	Developing supporting facilities for environmentally friendly mass transportation [99].
	Cooperating with organizations and industries related to transportation device designs, development, and the provision of supporting infrastructure and facilities [98].

## Data Availability

Not applicable.
